# Phytochemical and Biological Study of Trophic Interaction between *Pseudosphinx Tetrio* L. Larvae and *Allamanda Cathartica* L.

**DOI:** 10.3390/plants12030520

**Published:** 2023-01-23

**Authors:** Linda Matignon, Mame Marietou Lo, Magneric Monpierre, Mauro Vicentini Correia, Drochss Pettry Valencia, Marcos V. Palmeira-Mello, Marie-Noëlle Sylvestre, Ludovic Pruneau, Muriel Sylvestre, Antonio Domenech, Zohra Benfodda, Patrick Meffre, Gerardo Cebrián-Torrejón

**Affiliations:** 1 COVACHIM-M2E Laboratory EA 3592, Department of Chemistry, Fouillole Campus, University of the French West Indies, UFR SEN, CEDEX, 97157 Pointe-à-Pitre, France; 2 CHROME Laboratory, EA7352, University of Nîmes, CEDEX 1, 30021 Nîmes, France; 3 Institut de Systématique, Evolution, Biodiversité (ISYEB), Department of Chemistry, Fouillole Campus, University of the French West Indies, UFR SEN, CEDEX, 97157 Pointe-à-Pitre, France; 4 Instituto de Química, Campus Universitário Darcy Ribeiro, Universidade de Brasília, Brasília 70910-900, Brazil; 5 Departamento de Ciencias Naturales y Matemáticas, Pontificia Universidad Javeriana sede Cali, Calle 18 No. 118-250, Cali 760031, Colombia; 6 Instituto de Química, Universidade Federal Fluminense, Outeiro S. João Batista S/N, Niterói 24020-141, Brazil; 7 Departament de Química Analítica, Facultat de Química, Universitat de València, Dr. Moliner 50, 46100 Valencia, Spain

**Keywords:** chemical ecology, electrochemical ecology, aposematism, allelopathy, plant–insect interactions, *Pseudosphinx tetrio*, *Allamanda cathartica*, phytochemical analysis

## Abstract

In this article, we propose to explore the chemical interaction between *Pseudosphinx tetrio* L. and *Allamanda cathartica* L. using different analytical methods, including an innovative electrochemical approach (called electrochemical ecology) and multivariate analysis, and we investigate the potential antimicrobial effects (antibacterial and antifungal activities) of this interaction in order to gain a better understanding of their specific interaction. The analytical study presents a similar chemical profile between the leaves of healthy and herbivorous *A. cathartica* and the excretions of the caterpillars. The similar analytical profile of the leaves of *A. cathartica* and the excretions of *P. tetrio*, and the difference with the caterpillar bodies, suggests a selective excretion of compounds by the caterpillar. The measured antimicrobial activities support the physicochemical tests. The natural products found selectively in the excretions (rather than in the body) could explain the ability of *P. tetrio* to feed on this toxic Apocynaceae species.

## 1. Introduction

The tetrio sphinx, *Pseudosphinx tetrio* L. (Lepidoptera: Sphingidae), is a moth widely distributed in tropical and subtropical regions of the Americas and the Caribbean basin, where suitable host plants for the development of its larvae (large, conspicuous caterpillars) are present [1,2,3,4,5,6,7,8,9,10].

Adult females of *P. tetrio* [11] lay 50–100 eggs on the underside of the host plant’s leaves, from which they hatch in about three days [3]. After hatching, the gregarious larvae live in colonies for at least the first three developing stages, feeding initially on the upper surface of leaves and in later stages on all leaf tissues except leaf veins and midribs as they progress to the later instars. Adult larvae (fifth or sixth stages) feed singly or in small groups on entire leaves can reach a length of 63–69 mm [3,4]. The color of larvae is characteristic of aposematism (antipredation strategy: warning predators of bad taste or toxicity). According to an aposematic hypothesis, it becomes toxic to its predators when it ingests toxic phytocomponents from the milky sap of some plants [12]. The body is velvety black with yellow rings on each thoracic and abdominal segment, the head is dark orange-red, and the prothoracic shield, prominent legs and anal segment are orange-red with dark markings. The spine on the eighth abdominal segment is black and emerges from a raised, orange, button-like structure [4,13,14,15]. The aposematic larvae have few predators due to their venomous nature. However, the squirrel cuckoo, *Piaya cayana* Linn., of Belize, consumes *P. tetrio* by taking larvae, beating them against a branch until the poisonous gut contents are gone, and then swallowing the remains [16]. The larvae wrap themselves in a cocoon of silk or dead leaves and molt into new pupae about 7.0 cm long. The adult moths, colored in shades of gray, hatch from the pupae 53 days after egg laying and have a wingspan of 12.7–14.0 cm (eggs hatch in 3 days or more; average duration of the 5–6 larval stages is 23–4 days; the prepupal stage lasts about 4 days and the pupal stage about 22 days [3]). *P. tetrio* has the ability to eat up to twice its weight in food during a day, hence its name, “glutton caterpillar”. The larva of *P. tetrio* feeds preferentially on the leaves of the Apocynaceae family’s plants, such as *Allamanda cathartica* L. and *Plumeria alba* L., which are rich in toxic latex [17,18].

*A. cathartica* is commonly known as yellow allamanda, golden trumpet or “*Liane à lait*” [19]. This is a common name for the species. In the review paper by Ghosh and coworkers, it was described as a hermaphroditic, vine-like, woody shrub used primarily in landscaping, more spreading than tall, and with perennial leaves [20]. *A. cathartica* is a fast-growing species that is widely distributed worldwide, but mainly in tropical and subtropical regions, where the caterpillar *P. tetrio* is present [21,22]. *A. cathartica* is one of the fifteen species of the Apocynaceae family according to the “WFO Plant List” [23]. 

The plant is native to Brazil [24] and its various parts such as leaves, flowers, roots and stems are used in traditional medicine [25] for the treatment of jaundice [26], and have various pharmacological activities such as wound healing [26], antioxidant [27,28,29,30], antimicrobial [30,31,32], antifungal [33], antimalarial [34], anti-inflammatory [35]; anticancer [20,36,37] and gastrointestinal effects [38]. A dichloromethane (CH_2_Cl_2_) extract of the whole plant tested on two pathogenic dermatophytes, *Trichophyton rubrum* and *Microsporum gypseum*, showed moderate activity at a concentration of 50 μg/disk, but very strong activity at a concentration of 200 μg/disk, suggesting that *A. cathartica* may possess antidermatophyte constituents that could be useful in the treatment of ringworm [39,40].

In general, insects are not strictly restricted to the leaves and reproductive parts of living plants. Most studies conducted on strict herbivores [41] indicate that larval hosts of sphingids are concentrated on a few plant families whose foliage is particularly rich in alkaloids, milky sap, essential oils, irritant acids or other small toxic natural products (e.g., Rubiaceae, Euphorbiaceae, Apocynaceae, Vitaceae, Bignoniaceae, etc.).

However, all parts of *A. cathartica* are rich in secondary metabolites, which are exhaustively listed in the review by Petricevich and Abarca-Vargas [42], with plumieride, plumericin, and allamandin being the most characteristic. A total of 151 compounds were distributed as follows: 3 hydrocarbons in flowers; 7 alcohol compounds, 9 esters, 1 ether, 6 aldehydes, and 1 ketone in flowers, leaves, and stems extracts; 37 fatty acids and phospholipids; 43 volatile compounds mostly in flowers and leaves; 5 phenolic compounds and 6 flavonoids in the flowers and stems; 2 alkaloids in stems; 11 steroids and terpenes in leaves, stems and flowers; and 14 lactones in roots, stems, leaves, flowers and bark; 6 carbohydrates in leaves, stems, and nectar. Of the compounds identified, leaves contain: ursolic acid, β-amyrin; β-sitosterol, sesquiterpenes, plumericin, plumieride, long chain esters, flavonoids, polyphenols, allamandin, alkaloids, saponins and carbohydrates; stem and bark contain: ursolic acid, β-amyrin, β-sitosterol, triterpenoids, glucosides, alkaloids, flavonoids and polyphenols; flowers contain: quercetin, quercitrin, kaempferol, hesperetin, flavonoids, polyphenols and polysaccharides; and roots contain: lactones, allamandin, allamandicin, allamdin, plumieride iridoids, triterpenoids, alkaloids and several glucosides [36,38,43] (cited by [44]); [19,21,27,42]. Like *A. cathartica*, various parts of *P. rubra* are widely used in traditional medicine [45]. *P. rubra* contains many chemical constituents such as glycosides, phenolic compounds (phenolic acids and flavonoids), alkaloids, amino acids and terpenoids (iridoids) which give the flower antibacterial and antifungal activities. However, the main chemical constituents responsible for the pharmacological activities of *P. rubra* are: plumieride, fulvoplumierine, lupeol, rubrinol, stigmasterol, oleanolic acid, taraxasteryl acetate, rubranonoside, plumieride-p-E-coumarate, isoplumericin, rubrajalellol and plumericin [46,47,48,49]. These molecules form plant may have antimicrobial activities and effects and may be of interest for use against pathogenic microorganisms. 

In nature, predatory insects are highly host-specific and exhibit a variety of traits. Direct associations between plants and insects are ubiquitous, and in the context of tropical chemical ecology, many different plant–herbivore interactions are highly specialized [50]. What attracts an egg-laying insect to one plant and prevents it from laying its eggs on another is often an aspect of plant chemistry that is recognized by the insect [51]. Nevertheless, plants can biosynthesize protective secondary metabolites (in the milky sap or via vascular tissue or in specialized tissues) in a self-defense process to reduce herbivory. In turn, herbivores may respond to these compounds and attempt to metabolize them, selectively excrete them, or use them (after ingestion into the body) for their own defense [52,53]. In 2022, McCoy and colleagues showed that for Eocene herbivorous insects that eat leaves with such defense mechanisms, a distinct burrowing or cutting behavior that disrupts the supply of protective compounds distal to the plant tissue allowed the insect to eat the leaf [54].

The coevolution of specialized plant–insect herbivore interactions has been the subject of decades of study [55,56,57]. Lepidoptera caterpillars, for example, often specialize on toxic plants and are able to either sequester [58,59,60], metabolize [61,62] or excrete chemical compounds from their hosts in an unmodified form [63]. Understanding how herbivores deal with toxic phytochemicals in their diet is necessary to understanding the evolution of the two most biodiverse groups of multicellular organisms: plants and insects.

In this article, we propose to explore the chemical interaction between *P. tetrio* and *A. cathartica* using different analytical methods, including an innovative electrochemical approach (called electrochemical ecology) and multivariate analysis, and we investigate the potential antimicrobial effects (antibacterial and antifungal activities) of this interaction, in order to gain a better understanding of their specificity. 

## 2. Results and Discussion

### 2.1. Phytochemical Tests

***Thin layer chromatography (TLC).*** First, a TLC study was conducted. The different results of this approach are presented in Table 1. In our first hypothesis (Hyp. 1), we propose that the caterpillars take up and store certain metabolites (for example, the metabolite with an Rf (Retention factor) of 0.16 on TLC 3 (orange color)). The second hypothesis proposed (Hyp. 2), also listed in Table 1, refers to the selective excretion of an unidentified molecule by the caterpillars (for example, the metabolite with an Rf of 0.95 on TLCs 2 and 3 (blue color)). Finally, the third hypothesis (Hyp. 3) refers to the response of the healthy plant to herbivory. For example, the metabolite with an Rf of 0.28 on TLC 1 (pink color), since this metabolite is present in healthy leaves and not in predated leaves. The opposite case is observed with the metabolite with an Rf value of 0.18 on TLC 4 (green color), which is present only in predated leaves and could therefore be considered a metabolic marker for herbivores.

**Figure 1:**
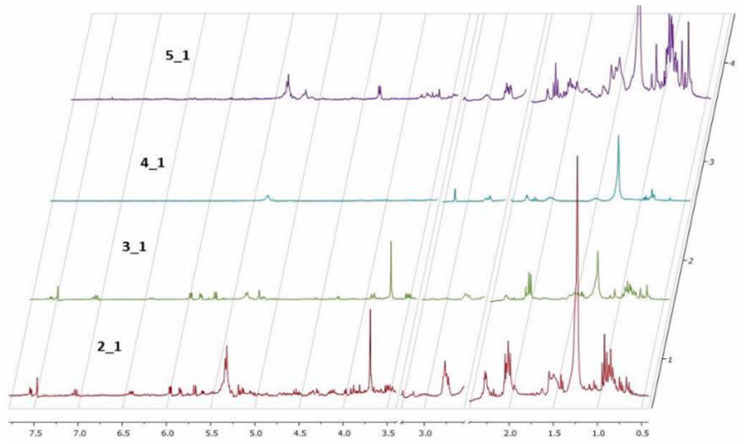
^1^H NMR of organic extracts in DMSO-d^6^. (2_1) Healthy *Allamanda* leaves; (3_1) predated *Allamanda* leaves; (4_1) caterpillar bodies and (5_1) caterpillar feces.

***Nuclear magnetic resonance spectroscopy^1^H NMR***. The ^1^H NMR data were obtained only from the organic extracts of the plants and caterpillars using DMSO-d^6^ as a solvent. Solvent signals from deuterated DMSO and residual water at 2.5 and 3.3 ppm, respectively, were removed (Figure 1). The analysis shows minor differences between the spectra of healthy and predated *Allamanda* leaves. This result is inconsistent with hypothesis 3 (in which a plant response to aggression was observed). 

The ^1^H NMR spectra of caterpillars’ bodies and feces show large differences between the samples, and the chemical profile of healthy *Allamanda* leaves and the caterpillar feces is very similar. This result confirms hypothesis 2 and shows the similarity between the chemical profile of *A. cathartica* leaves and caterpillar feces compared to the caterpillar body. These similarities and dissimilarities confirm our hypothesis (Hyp. 3) of the selective excretion of toxic compounds. 

**Figure 2:**
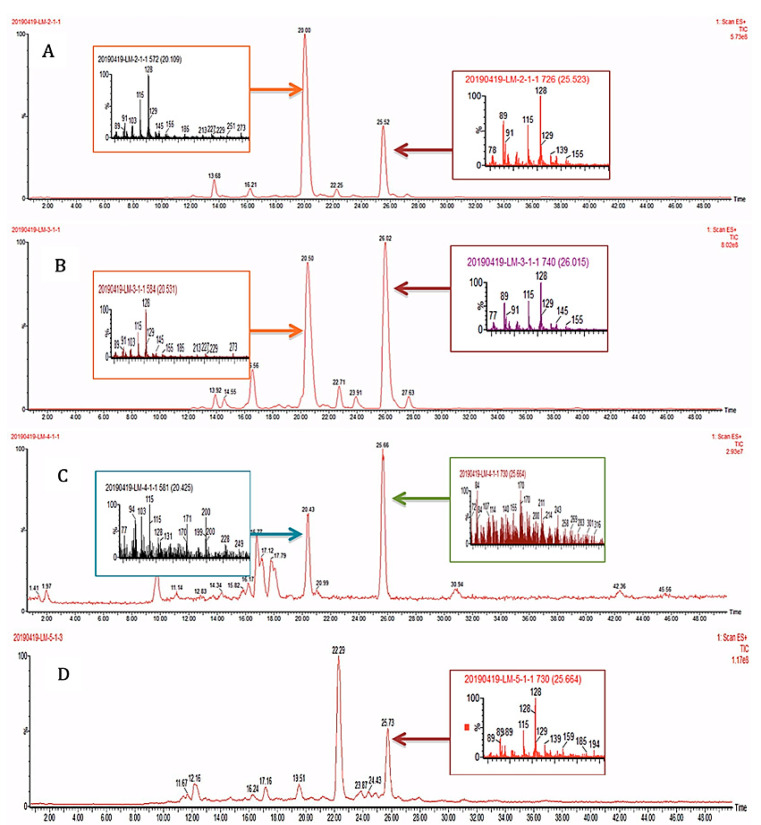
HPLC-MS profiles of (**A**) healthy and (**B**) predated leaves of *A. cathartica* and (**C**) caterpillar bodies and (**D**) caterpillar feces.

***High performance liquid chromatography coupled with mass spectrometry (HPLC-MS).*** In this study, the samples containing the healthy and eaten leaves of *A. cathartica*, as well as the caterpillars and their feces, were analyzed using high-performance liquid chromatography techniques [19,64,65,66] (Figure 2). The similar profile observed in these spectra indicates the presence of the same main compounds in the healthy (A) and predated (B) *A. cathartica* leaves, with similar retention times (RT) of 20.00 min and 20.50 min, respectively. A second compound can be identified in these spectra with comparable retention times to hypothesis 3 (RTs: 25.52 min and 26.02 min). These results are in contrast to hypothesis 3 (as we can see in the TLCs and ^1^H NMR profiles). The analysis shows the presence of this compound in the caterpillar’s feces (D, RT: 25.73 min) but not in its body (C), which also confirms hypothesis 2 (in agreement with the ^1^H NMR and TLC results). Finally, the results show that the compounds found in *P. tetrio*’s bodies (C, RT: 20.43 min and 25.66 min) are significantly different from other compounds found in the feces (also confirming hypothesis 2).

***Electrochemical investigation.*** Cyclic voltammetry (CV) studies have been used to investigate possible interactions between different compounds and were previously used to monitor plant defenses against external stressors [67,68,69,70]. Here, the predation of *A. cathartica* by *P. tetrio* has been monitored electrochemically using CV. We studied the electrochemical response of films (on the surface of a glassy carbon electrode) of organic and aqueous extracts of healthy and predated leaves of the plant and samples of bodies and feces of *P. tetrio* in 0.10 mol L^−1^ phosphate solution buffer at pH 7.0. The reported methodology is ultimately based on the voltammetry of microparticles (VMP) technique [71]. 

**Figure 3:**
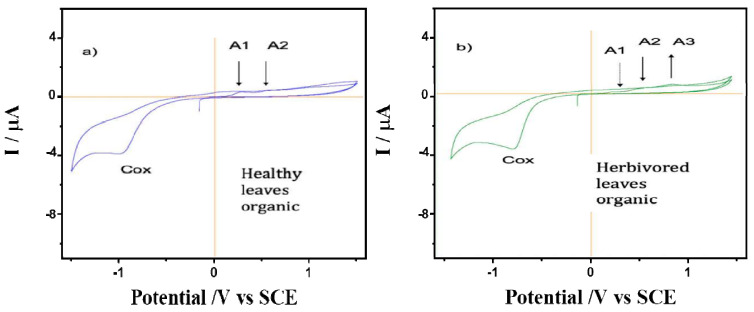
Cyclic voltammograms of films from (**a**) healthy and (**b**) predated leaves of *A. cathartica* on glassy carbon electrode in contact with air-saturated 0.10 M phosphate buffer at pH 7.0. Scan rate 10 mV.s^−1^.

The behavior of films on glassy carbon electrodes of healthy and predated leaves of *A. cathartica* is shown in Figure 3. A series of chemically irreversible oxidation processes: A_1_, A_2_ and A_3_, were observed (0.28 V, 0.65 V and an undefined shoulder at 1.00 V, respectively). While the peaks A_1_ and A_2_ decrease with cycling until a fully passivated and stable state is reached at about the second cycle, an increase in the A_3_ peak was observed. Thus, the variations in the ratio of peaks A_1_ and A_3_ suggest differences in the nature of the redox species of *A. cathartica* and in leaves that are strengthened by them. These oxidation processes represent a profile observed in several plant species and are due to the oxidation of polyphenolic organic compounds. Indeed, the voltammogram of the predated leaves exhibits the same signals as healthy leaves, but peak A_1_ is depleted while the A_3_ signal is enhanced. Finally, a reduction process can be observed at about −1.0 V when the potential sweep is reversed to less positive potentials. Although these processes are slightly more negative in healthy leaves than in predated leaves, both are consistent with the typical behavior of the oxygen reduction reaction (ORR) in water. This result basically contradicts hypothesis 3 about the chemical response to the herbivory, but using this technique, we can identify slight differences between healthy and predated leaves, possibly related to polyphenols (reaction against oxidative stress) [72].

**Figure 4:**
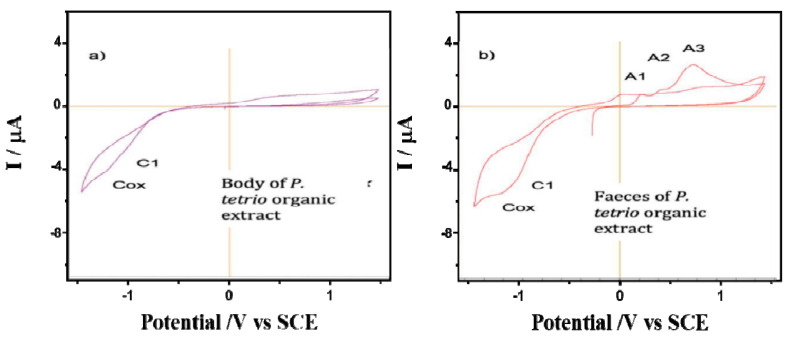
Cyclic voltammograms of films from (**a**) *P. tetrio*’s bodies and (**b**) *P. tetrio*’s excrement after predation on leaves of *A. cathartica* on glassy carbon electrode in contact with air-saturated 0.10 M phosphate buffer at pH 7.0. Potential scan rate 10 mV.s^−1^.

The CV of films on glassy carbon electrodes of *P. tetrio*’s bodies and its excrement after predation of leaves of *A. cathartica* are shown in Figure 4. CV shows no clear oxidation process in the initial anodic scan for the body of *P. tetrio*, but three oxidation processes for its excreta at 0.22, 0.45 and 0.78 V for A_1_, A_2_ and A_3_, respectively, which are similar to those found for the *A. cathartica* analysis. Again, a typical oxygen reduction reaction (ORR) in water was observed in the cathodic region in both analyses. In addition, a characteristic signal C_1_ appears in both samples, indicating the presence of oxidized metabolite of *P. tetrio*. This result confirms hypothesis 2, in agreement with the other analytical approaches.

**Figure 5:**
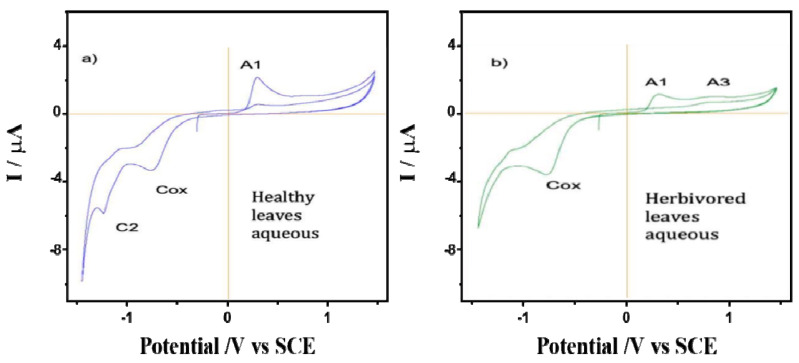
Cyclic voltammograms of films from the water extracts of (**a**) healthy and (**b**) predated leaves of *A. cathartica* on glassy carbon electrode in contact with air-saturated 0.10 M phosphate buffer at pH 7.0. Potential scan rate 10 mV.s^−1^.

**Figure 6:**
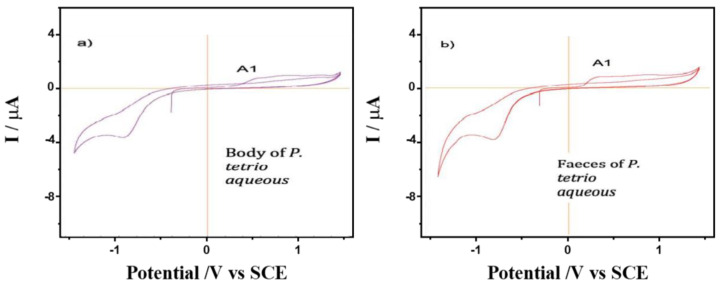
Cyclic voltammograms of films from water extracts of (**a**) *P. tetrio*’s bodies and (**b**) its excrement after predation on leaves of *A. cathartica* on glassy carbon electrode in contact with air-saturated 0.10 M phosphate buffer at pH 7.0. Potential scan rate 10 mV.s^−1^.

A comparison was made between the CV of films from the water extracts of healthy and spoiled leaves of *A. cathartica* in contact with air-saturated 0.10 mol L^−1^ phosphate buffer at pH 7.0 (Figure 5). Here, signal A_1_ appears clearly recorded in the healthy leaves and is accompanied by a well-defined cathodic peak at—1.25 V (C_2_). Signal C_2_ disappears in the CV of the damaged leaves, while signal A_1_ decreases and signal A_3_ is clearly visible at 0.78 V. This result confirms hypothesis 3, which shows a reaction of leaves against herbivory; the disappearance of signal C_2_ and the appearance of signal A_3_ are a result of herbivory. This CV is reproduced to some extent reproduced in the aqueous extracts of *P. tetrio* and its excrement, as shown in Figure 6, thus suggesting the existence of common electroactive compounds.

**Figure 7:**
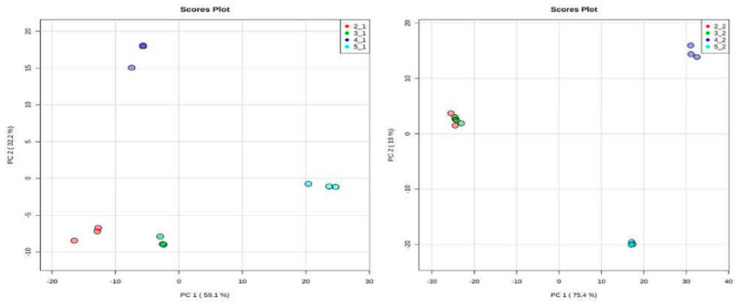
Score plot of 12 samples of organic and aqueous extracts of all manipulation, obtained from 168 (**left**) and 412 (**right**) variables, respectively. (2_1and 2_2) red circle, healthy *Allamanda* leaves; (3_1and 3_2) green circle, predated *Allamanda* leaves; (4_1and 4_2) blue circle, caterpillar bodies and (5_1and 5_2) light blue circle, caterpillar feces.

**Figure 8:**
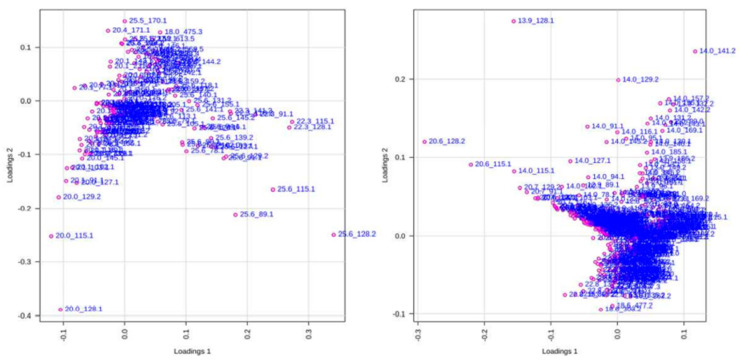
Score plots of 12 samples of organic and aqueous extracts of all manipulation, obtained from 168 (**left**) and 412 (**right**) variables, respectively. Each dot represents one variable (retention time m/z).

***Principal Component Analysis (PCA).*** A total of 24 chromatograms were acquired using HPLC-EI-MS, 12 from the aqueous fraction and 12 from the organic fraction (three biological replicates for each species). To identify differences in the metabolites or even in their concentration in the samples, the set of 24 samples was analyzed, obtained from XCMS and used for data analysis. Two spreadsheets were obtained after data processing of the HPLC-MS analysis, with 168 and 412 variables from the organic and aqueous fractions, respectively (retention time m/z). In the PCA analysis, two plots were generated, namely score and loading plots. The first one shows the sample groupings, whereas the second one indicates the contribution of each variable to these samples. The PCA analysis was focused on the detection of any inherent pattern within the data. As observed in Figure 7, samples of healthy and predated *Allamanda* leaves are closely related, with small differences from each other, when compared with the caterpillars’ bodies and feces. Further, when all samples are compared, the predated and not predated leaves from *Allamanda* are very similar to each other, and different from the other samples. In addition, the caterpillar body extracts present a great difference. In Figure 8 (loadings plot), the variables that influence the grouping observed are presented.

### 2.2. Microbial Activities

The potential antimicrobial activities of *P. tetrio*/*A. cathartica* interaction are shown in Table 2. The yellow boxes show the effects of the consumption of the *A. cathartica* leaves by the caterpillar. The comparison between the healthy leaves (A) and the leaves eaten by the caterpillar (B) shows an increase or occurrence of inhibitory activity in favor of the leaves eaten by the caterpillar (B) on the microorganisms, regardless of the type of extraction (Aqueous (_A_); Organic (_O_)), A_O_ versus B_O_ for *Escherichia coli* and *Candida albicans* (diameter of inhibition: 7 mm versus 0 mm; 9,5 mm versus 7 mm); and A_A_ versus B_A_ for *Escherichia coli* and *Candida albicans* (diameter of inhibition: 7 mm versus 0 mm; 9 mm versus 8 mm)). The occurrence or enhancement of this bioactivity in the predated leaf could be explained by the induction of a chemical or biological defense reaction of the host plant against herbivory (proposed hypothesis 3). 

The green and blue boxes, respectively, represent the non-uptake and the uptake of bioactive compounds from the leaves into the bodies of the caterpillars. The samples obtained after extraction with organic solvents (_O_) show the same evolution of the inhibitory profile in three of the five microorganisms tested (*Escherichia coli, Staphylococcus aureus* and *Aspergillus fumigatus*). Our results suggest a selective excretion of bioactive compounds from ingested leaves into the feces without being retained or ingested in the caterpillar’s body (green boxes). Indeed, the feces of *P. tetrio*’s (D_O_) show inhibitory activity that is as strong or stronger than that of the eaten *A. cathartica* leaves (B_O_) [diameter of inhibition fecal matter (D_O_) versus eaten leaves (Bo): 11 mm versus 7 mm for *Escherichia coli;* 9.5 mm versus 8 mm for *Staphylococcus aureus;* and 10,5 mm versus 11 mm for *Aspergillus fumigatus*) whereas caterpillar body extracts (C_O_) showed no bio-inhibition (proposed hypothesis 2). The inhibitory effect of the organic and aqueous samples on *Pseudomonas aeruginosa* and *Escherichia coli*, respectively (blue boxes), shows an opposite profile. Indeed, the eaten leaves and the caterpillar body show a bio-inhibition that is not found in the caterpillar feces. This suggests that certain bioactive compounds in the eaten leaves, different from the previous ones, could be selectively taken up and stored specifically in the caterpillar body.

There are few data in the literature on the potential antimicrobial activity of predated leaves and the interaction between caterpillars and plants. Our samples showed higher antimicrobial activity than that reported by Islam et al. (2010) [31] for a concentrated methanol extract of healthy *A. cathartica* leaves fractionated into fractions soluble in petroleum ether, carbon tetrachloride, chloroform and water. The zones of inhibition observed by them have a diameter of less than 10 mm for *E. coli*, *S. aureus*, *C. albicans* and *P. aeruginosa*, at a tested concentration of 400 µg disk^−1^ against the 100 µg disk^−1^ in our case. At the same time, they showed higher antimicrobial activity with their methanolic and ethyl acetate extracts from healthy leaves [73]. Quercitrin was explicitly attributed antifungal and antibacterial activity [43]. 

Our qualitative results on biological activity may explain why *P. tetrio* can feed on this toxic Apocynaceae species. Indeed, the spectral (NMR) profiles of the eaten leaves and the caterpillar droppings are very similar, but very different from those of the caterpillar bodies. There is an association between the plant and caterpillar (specific herbivory). The plant produces organic chemical compounds for defense when the caterpillar attacks, and the caterpillar excretes or assimilates these molecules or derivatives by its own mechanism. The inhibition may be due to a synergistic effect of the different molecules contained in the extracts for all the microorganisms, hence the interest in using different analytical techniques. The genetic and cellular mechanisms by which metabolite diversity arises are becoming better understood, but the evolutionary explanations for the continued diversification of plant secondary metabolites have received less attention. Speed et al. (2019) [74] show a fundamental coevolutionary asymmetry between plants and their herbivores, which is that herbivores must resist all plant toxins, whereas plants must challenge and override only a single resistance trait.

Sequestration likely evolved as a protection from predators [75], [76] because caterpillars with chemical protection are less attractive to predators [77]. *P. tetrio* feeds on toxic species of the Apocynaceae and is aposematically colored, leading to the hypothesis that *P. tetrio* caterpillars may sequester compounds from their host. In many plant species, herbivory leads to the increased production of a secondary metabolite [78]. In particular, herbivores specialized in sequestration are thought to benefit from the induction of intermediate levels of chemical defenses, while plants eaten by such herbivores would benefit most from weak or strong induction. On the other hand, specialists that do not specialize in sequestration may not be deterred by induction unless it is high [55]. Caterpillars also have the ability to metabolize some organic chemical compounds and excrete others intact from the same host plant [63]. This is the case in Monarch butterflies, *Danaus plexippus* Linn. [58]. The study of Ramos et al., 2015 [79], compared the proteolytic system of the gut of *P. tetrio* and *D. plexippus* with the proteolytic system of the milky sap of their respective host plants (*P. rubra* and *Calotropis procera* Aiton). This revealed that the ability of the insect proteolytic systems (serine and cysteine peptidase inhibitors) to digest the milk sap proteins (in vivo) appears to be an important event favoring the caterpillars in overcoming plant chemical defenses. 

## 3. Materials and Methods

### 3.1. Plant Material

***Sample preparation.*** Healthy and eaten leaves of *A. cathartica*, as well as *P. tetrio* caterpillars were collected on the island of Guadeloupe (French West Indies), specifically in Le Gosier (16°13’00.5” N 61°31’09.9” W). The leaves were cleaned (with distilled water) and lyophilized. The caterpillars were kept in a cage for 24 h, to collect their feces, and starved for 48 h to use their corpse in the evaluation of biomolecule incorporation into the bodies of the caterpillars, excluding the digestive material. Finally, the dried leaves, the caterpillars’ bodies and feces were lyophilized and powdered. Afterwards, 50 g of each sample was extracted by maceration extraction for 48 h. Maceration was performed in a ternary mixture of dichloromethane/methanol/distilled water [80,81] (1:1:1 *v/v*, 200 mL). After the complete extraction, the solutions were filtered and extracted by liquid–liquid extraction and two phases were obtained for each manipulation (Table 3). Finally, these phases were dried by rotary evaporation. 

### 3.2. Phytochemical Tests

***Analysis and quantification.*** Analyses were performed using several techniques such as Thin Layer Chromatography (TLC), Nuclear Magnetic Resonance Spectroscopy (1H-NMR), High Performance Liquid Chromatography coupled with Mass Spectrometry (HPLC-MS) and Cyclic Voltammetry (CV). 

***Thin layer chromatography (TLC).*** The thin layer chromatography of all the organic phases was carried out with a gradient of two different mixtures of elution solvents composed of CH_2_Cl_2_/MeOH or nhexane/AcOEt with a volume of 10 mL in different proportions (80/20, 50/50, 90/10, 95/5, 99/1, 50/50). Further, TLCs of all aqueous phases were made on cellulose TLC with a mixture of H2O/MeOH elution solvent in different proportions (50/50–100) as a mobile phase.

***Nuclear magnetic resonance spectroscopy (1H-NMR).*** 15 mg of each extract was solubilized in 650 µL of DMSO and transferred to NMR tubes. 1D 1H nuclear magnetic resonance spectra were recorded with a BRUKER Avance 300 MHz spectrometer equipped with a BBO probe and automatic tube changer. Chemical shifts (δ) were expressed in ppm relative to tetramethylsilane (TMS) taken as the external reference, with internal calibration performed on the solvent signal. All spectra were processed using Topspin 2.1 software. The classical 1D proton with a 90° pulse width was performed. The spectra were acquired using 256 scans and 2 dummy scans of 32 K data points with a spectral width of 5411.255 Hz. 

***High performance liquid chromatography coupled with mass spectrometry (HPLC-MS).*** Triplicates of each extract were made and dissolved in 750 µL of methanol (LCMS grade) and 750 µL of ultra-pure water (ElgaPurelab Classic). The mixtures were sonicated in the ultrasonic bath then filtered on 13 mm and 0.45 µm PTFE filters and placed in amber vials. The analysis was performed on a Waters (Milford, MA, USA) Alliance e2695 liquid chromatographic system, equipped with a Waters 2996 photodiode array detector (PDA), coupled with a orthogonal quadrupole mass spectrometer (Micromass ZQ, Manchester, UK). The systems were controlled by MassLynx v.4.1 software (Micromass, Manchester, UK). The mass was equipped with an electrospray ionization ESI (Waters) source; the ionization was performed in positive mode. A double detection was carried out via mass spectrometry in ESI (range 71 to 1200 Da) and using a PDA diode array detector (UV detection between 210 and 400 nm). The analytical column was an XTERRA MS C18 column (2.1 x 100 mm, 3.5 µm) (Waters). The elution was performed with a mobile phase flow rate of 0.2 mL/min consisting of a mixture of ultra-pure water containing 0.1% formic acid (A) and methanol containing 0.1% formic acid (B). The program started at t = 0 min with a ratio (A: B) of 95:5, at t = 2 min (95:5), at t = 5 min (70:30), at t = 15 min (60:40), at t = 40 min (50:50), at t = 55 min (45:55), at t = 60 min (0:100), at t = 65 min (0:100), at t = 75 min (95:5) and at t = 80 min (95:5). The injection volume was 10 µl and detection was at 280 nm and at 320 nm. The ESI-MS parameters were as follows: desolvation gas (N_2_) flow rate: 650 L/h; cone gas flow rate: 40 L/h; drying gas temperature: 450 °C; source temperature: 120 °C; capillary voltage: 3 kV; cone voltage: 85 V; and RF lens voltage: 0.1 V. 

***Cyclic Voltammetry (CV).*** Cyclic voltammograms were recorded at 298 ± 1 K in a conventional three electrode cell using a platinum wire auxiliary electrode and an Ag/AgCl (3M NaCl) reference electrode. Measurements were carried out with CH I660 equipment using 0.10 M potassium phosphate buffer at pH 7.0 as a supporting electrolyte. The working electrode was prepared by evaporating 50 μL of an ethanol (EtOH) suspension of the extract of interest, ground leaves or insect samples, under air on a glassy carbon electrode (GCE, BAS MF 2012, geometrical area 0.071 cm^2^). In order to mimic the natural environment, no degasification of the electrolyte was performed.

***Principal Component Analysis (PCA).*** The raw data from HPLC-MS were exported as netCDF files, using DataBridge software (Waters, USA), and pre-processed using XCMS Online [82,83] for feature detection, retention time correction and alignment of metabolites detected on HPLC-MS analysis. The dataset was created with 12 samples from each organic and aqueous extract fraction (3 samples from each manipulation (see Table 1)). Peak detection was performed using cent Wave peak detection (Δm/z = 10 ppm; minimum peak width, 5 s; maximum peak width, 20 s) and mzwid = 0.015, minfrac = 0.5, and bw = 5 were used for the retention time alignment. The processed data (csv file) were further exported to MetaboAnalyst 4.0 [84]. All data variables were scaled by the pareto method prior to PCA. PCA is an unsupervised method commonly used to identify patterns between multivariate samples [85,86] and was recently employed on the chemical variability of Allamanda cathartica extracts [87].

### 3.3. Antimicrobial Activities

To investigate the potential antimicrobial effects of the interaction between the caterpillar *Pseudosphinx tetrio* L. and one of its host plants, *Allamanda cathartica*, we focused our study on five pathogen microorganisms that are responsible of severe human disease and usually involved in hospital acquired illness: *Escherichia coli*, *Pseudomonas aeruginosa* and *Staphylococcus aureus* for bacteria and *Candida albicans* and *Aspergillus fumigatus* for fungi. All strains were supplied by the ATCC (American Type Culture Collection).

The three bacteria were cultured on the non-selective medium Tryptic Soy Agar, whereas the two fungi were cultured on the selective medium Sabouraud, which is a selective medium for fungi and yeasts. These two media were provided by the manufacturer Grosseron, France, and prepared according to the instructions.

For each microorganism, starting cultures were prepared on Tryptic Soy Agar media for bacteria and Sabouraud media for fungi. From these starting cultures, microbial suspensions were created by picking two colonies from the starting cultures and adding them to 2 mL of sterile water. Finally, 200 µL of these microbial suspensions were used to inoculate the culture media used to test the antimicrobial activities.

Disc diffusion assay was used in order to test the antimicrobial activities of interaction between *P. tetrio* and *A. cathartica*. Sterile filter paper discs of Whatman no.1 (6 mm in diameter) were placed on the surface of the culture media with sterile forceps and gently pressed to ensure good contact with the surface. The different organic extracts were prepared as follows: 5 mg of each extract was mixed in 500 µL of Dimethyl sulfoxide (DMSO) and 10 µL of these mixtures were placed on antibiotic disks (100 μg/disk). The plates were incubated for 24 h to 48 h at 37 °C for bacteria and 30 °C for fungi. The zone of inhibition was calculated by measuring the diameter of the inhibition zone around the well (mm). All tests were conducted in duplicate.

## 4. Conclusions

In conclusion, we investigated the plant–herbivore interaction between the caterpillar *Pseudosphinx tetrio* and the flowering plant *A. cathartica*. In order to better understand this trophic relationship, several techniques were used, such as TLC, 1H NMR, HPLC-MS (analyzed using a 13 multivariate PCA) and an innovative approach using electrochemical methods (electrochemical ecology). The measured antimicrobial activities support the physicochemical tests. The results show a similar profile between the leaves of healthy and predated *A. cathartica* and the excretions of the caterpillars. The similar analytical profile between the leaves of *A. cathartica* and the excretions of *P. tetrio*, and the difference with the caterpillar bodies, suggests a selective excretion of compounds by the caterpillar (proposed hypothesis 2). These organic compounds found selectively in the excretions (rather than in the body) could explain the ability of *P. tetrio* to feed on this toxic Apocynaceae species.

## Figures and Tables

**Table 1:** Organic phases obtained with TLC results. (A) Healthy *A. cathartica* leaves, (B) herbivored *A. cathartica* leaves, (C) caterpillar bodies and (D) caterpillars’ feces. Solvent percentages are presented as highest polarity in solution (MeOH and EtOAc) *.

Elution Solvent	% *	TLC	R_f_	A (1.2)	B (1.3)	C (1.4)	D (1.5)
CH_2_Cl_2_	100	1	0.16				
			0.28	Hyp. 1 and 3		Hyp. 1	
			0.58				
CH_2_Cl_2_/MeOH	5	2	0.05				
			0.19				
			0.22				
			0.60				
			0.91				
			0.95	Hyp. 2	Hyp. 2		Hyp. 2
	10	3	0.16	Hyp. 1	Hyp. 1	Hyp. 1	
			0.20				
			0.57				
			0.90				
			0.95	Hyp. 2	Hyp. 2		Hyp. 2
	1	4	0.18		Hyp. 1 and 3	Hyp. 1	
			0.30	Hyp. 1 and 3		Hyp. 1 and 3	
			0.79				
			0.93				
			0.94		Hyp. 2 and 3		Hyp. 2
CH_2_Cl_2_	100	5	0.08				
			0.17				
			0.37				
			0.62				
			0.93				
C_6_H_14_	100	6	0.03				
			0.37	Hyp. 1	Hyp. 1	Hyp. 1	
C_6_H_14_/C_4_H_8_O_2_	20	7	0.17				
			0.30				
			0.34	Hyp. 1	Hyp. 1	Hyp. 1	
			0.64				
			0.94		Hyp. 2 and 3		Hyp. 2
			0.97				
			0.98				
			1				
	50	8	0.48		Hyp. 1 and 3	Hyp. 1	
			0.95				
			0.97				
	80	9	0.50				
			0.91				
			0.94				

* Similarly, metabolites between two extracts or more are described in colors: orange: A, B and C; blue: A, B and D; pink: A and C; yellow: B and D. Hypotheses that can be illustrated by each compound are cited.

**Table 2:** Microbial activity results of aqueous and organic extracts, obtained from *Allamanda cathartica* (*A. cathartica*) leaves, caterpillar bodies and caterpillar feces on three bacterial (*Escherichia coli*; *Staphylococcus aureus*; *Pseudomonas aeruginosa*) and two fungal (*Candida albicans*; *Aspergillus fumigatus*) strains.

Extracts (100 µg/disc) Microorganisms	Caterpillar Feces (D)		Caterpillar Bodies (C)		Healthy *A. cathartica* Leaves (A)		Predated *A. cathartica* Leaves (B)	
	D_A_	D_o_	C_A_	C_o_	A_A_	A_o_	B_A_	B_o_
*Escherichia* *Coli*	-	+++ (11)	+ (7)	-	-	-	+ (7)	+ (7)
*Staphylococcus aureus*	-	++ (9.5)	-	-	-	+ (8.5)	-	+ (8)
*Pseudomo*- *nas aeruginosa*	-	-	-	++ (9)	-	+ (7)	-	+ (8,5)
*Candida* *albicans*	++ (8,5)	++ (9)	+ (7)	+ (7)	+ (8)	+ (7)	++ (9)	++ (9,5)
*Aspergillus* *fumigatus*	-	+++ (10,5)	-	-	-	+++ (10)	-	+++ (11)

*Legend:* Aqueous extracts (_A_); organic (_O_) extracts; healthy *A. cathartica* leaves (A); predated *A. cathartica* leaves (B); caterpillar bodies (C); caterpillar feces (D), on three bacterial (*Escherichia coli*; *Staphylococcus aureus*; *Pseudomonas aeruginosa*) and two fungal (*Candida albicans*; *Aspergillus fumigatus*) strains. (mm): size of the inhibition halos in millimeters. (-) no inhibitory activity; (+ to +++) more or less intense inhibitory activity.

**Table 3:** Phases obtained for each manipulation after liquid–liquid extractions.

	Organic Phase	Aqueous Phase
Healthy *A. cathartica* leaves	1.2	2.2
Hervivored *A. cathartica* leaves	1.3	2.3
Caterpillars	1.4	2.4
Caterpillar feces	1.5	2.5

## Data Availability

Not applicable.
